# Inspiratory Muscle Training with Isokinetic Device to Help Ventilatory Weaning in a Patient with Guillain-Barré Syndrome by Zika Virus

**DOI:** 10.1155/2018/9708451

**Published:** 2018-10-04

**Authors:** Leonardo Cordeiro de Souza, Amarildo Abreu de Souza, Eric Eduardo Pinto de Almeida, Leo Honse Ribeiro, Marcos David Parada Godoy, Wanderlei Augusto Junior, Jocemir Ronaldo Lugon

**Affiliations:** ^1^Medical Science Post-Graduation Program, Universidade Federal Fluminense, Niterói, RJ, Brazil; ^2^Intensive Care Division, Hospital e Clínica São Gonçalo, São Gonçalo, RJ, Brazil; ^3^Nephrology Division, Universidade Federal Fluminense, Niterói, RJ, Brazil

## Abstract

Zika is an arbovirus infection mainly transmitted by the mosquito* Aedes aegypti*. In 2016, the burden of Zika epidemic in Brazil was significant. Patients affected by Zika virus can develop Guillain-Barré syndrome, evolving to muscle respiratory failure requiring mechanical ventilation. In this setting, delayed recovery of the muscle weakness can result in prolonged weaning, a condition that by itself is related to a high mortality rate. The study is reporting a case of a patient with Zika and Guillain-Barré syndrome who underwent an inspiratory muscle training program starting after twenty-five days of mechanical ventilation and guided by serial measurements of the timed inspiratory effort (TIE) index. The patient was successfully weaned in two weeks and discharged from the hospital 30 days after extubation.

## 1. Introduction

In recent years, Brazil has witnessed an outbreak of new diseases transmitted by the mosquito* Aedes aegypti*. The emergence of Zika virus (an arbovirus of the Flavivirus genus) took place in northeast part of Brazil in 2015 and was followed by a rapid countrywide spread. By 2016, the numbers of Zika epidemics in Brazil were alarming: according to the Ministry of Health, the Zika virus reached more than 200 thousand people [1].

Guillain-Barré syndrome (GBS) is a neurological syndrome of autoimmune origin, characterized by an acute inflammatory polyradiculoneuropathy, which progresses rapidly, triggering an ascending symmetrical muscular weakness. This acute demyelinating neuropathy, which comprehensively affects the peripheral nervous system, carries a potentially fatal course [1].

Patients with Guillain-Barré syndrome related to the Zika virus may have respiratory muscle dysfunction, rapidly evolving to acute respiratory failure requiring the use of mechanical ventilation (MV). The delayed recovery of the muscle weakness can result in prolonged weaning, a condition that by itself is associated with a high mortality rate [1–3].

Inspiratory muscle training (IMT) with linear pressure loading devices is the major current therapeutic expectation in the quest for ventilator independence. Since 2002, several studies have demonstrated that the use of IMT may increase the success rate of the weaning process [2, 4]. To improve these numbers the timed inspiratory effort (TIE) index was incorporated to compose the tools used to assess and guide the recovery of respiratory muscle dysfunction.

This is a report of a case of a Zika-related Guillain-Barré syndrome in prolonged mechanical ventilation that underwent an IMT program with an isokinetic electronic load device. The adequacy of the inspiratory muscle strength was assessed and guided by serial measurements of the timed inspiratory effort (TIE) index, recently reported as a useful tool to help in the mechanical ventilation weaning.

## 2. Methods

The patient was cared for in a hospital located in the city of São Gonçalo, in the state of Rio de Janeiro, Brazil, from June 28^th^, 2017, to August 22^nd^, 2017. A free and informed signed consent form was obtained for the study.

### 2.1. Measurement of the TIE Index

A recently proposed timed inspiratory effort (TIE) index, which integrates the PImax with the time to reach it, showed better performance than the rapid and superficial respiration index (f/Vt), maximal inspiratory pressure (PImax), respiratory drive (P0.1), and integrative weaning index (IWI). The TIE index is calculated as the ratio of the PImax registered after the first 30 seconds of observation by the corresponding time to reach it while keeping the airways occluded with a unidirectional valve for up to 60 seconds [2].

For the measurement of maximal inspiratory pressure (MIP) and TIE index we used a prototype of a digital vacuometer called MagnaTIE (Magnamed, São Paulo, SP, Brazil), with a scale of 300 cmH_2_O and an increment of 0.1 cmH_2_O and a time interval of 100 ms for each pressure.

As a safety measure during the use of the TIE index, the subject of the study was kept under continuous surveillance through the multiparameter monitor (Dixtal, São Paulo, SP, Brazil); if any sign of instability was noticed by the examiner, the test was stopped and the patient returned to MV.

The method used was the occlusion of the airway with the unidirectional valve. The patient was positioned in dorsal decubitus, with the head elevated to 45 degrees. The cuff was hyperinflated to prevent leakage during measurement. After tracheal aspiration, patient remains connected to the mechanical ventilator for rest for two minutes with 100% oxygen inspired fraction (FiO2).

After the hyperoxygenation, mechanical ventilator disconnection was performed and after 10 seconds of spontaneous breathing, the digital vacuometer was connected manually in the end of an expiration to the artificial airway, keeping the airways occluded for up to 60 seconds and recording the values corresponding to each inspiratory effort. Thereby, the TIE index was calculated with the ratio of the maximum inspiratory pressure registered after the first 30 seconds of observation and the corresponding time to reach it [2].

Weaning success was predicted by values of the TIE index ≥1.0 cmH_2_O/s, and the patient uninterruptedly remained on spontaneous breathing for 5 days [5]. The decision to return to mechanical ventilation was made by a respiratory physiotherapist and the physician in charge, based on the signs of poor tolerance used routinely in the hospital [2].

**Figure 1:**
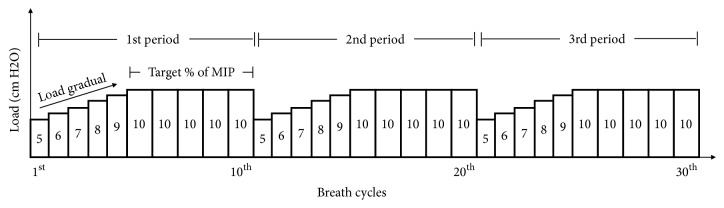
Diagram of the protocol in 3 load cycles each (in each inspiratory muscle training session the procedures were performed twice).

The IMT was conducted daily between 08:00 and 10:00 am. The established intermittent protocol consisted of imposing an inspiratory load for 30 breath cycles in three periods (Figure 1). Each period of 10 breath cycles was divided two steps: in the first, the load was gradually increased until the target was reached (45% of the MIP); in the second, the remaining 5 breath cycles of the period were run under the target load. At the end of the third period, patients had one period of rest (breathing without inspiratory load) of 2-3 minutes and underwent three more periods of training totaling 6 periods (60 breath cycles) of training each time. If signs of intolerance were observed during the IMT the patient was returned to MV. After each training session, the patient was returned to the MV in PSV mode for two hours to rest, a protocol of progressively lengthening tracheal collar trials, which increased in length each day [5]. The patient was reassessed weekly for the TIE index.

## 3. Case Report

A 33-year-old male, without comorbidities, was admitted to the hospital on June 28^th^, 2017, reporting fever (39.5°C) and paresthesia in the feet upwards. He had difficulty in walking and speaking; he also related episode of the fall from his own height. He was admitted to the intensive care unit (ICU) with dyspnea, pneumonia, and complaints of persistent muscular pain. His initial laboratory tests showed hemoglobin 14.0 g/dL, white blood count 6.780/mm^3^, BUN 8.4 mg/dL, creatinine 0.72 mg/dL, Na 143mEq/L, K 4.2 mEq/L, CK 138U/L, and PCR 10.1mg/dL. On the second day of admission, serology was positive for Zika virus (IgG antibodies).

On June/30/2017, he developed acute respiratory insufficiency type II, requiring sedation and orotracheal intubation and was placed on mechanical ventilation with pressure controlled mode (PCV). Due to the severity of his clinical condition, he was maintained with this regimen until the beginning of the weaning process. His cerebrospinal fluid showed an increased presence of proteins (191mg/dL) with a normal cell count consistent with Guillain-Barré syndrome. Blood cultures for bacteria or fungi and serological tests for toxoplasmosis, herpes, varicella, and HIV were all negative. During his stay in the ICU, 0.4g/kg intravenous immunoglobulin was administered for 5 days without improvement. In the same period, the patient developed septic shock of undetermined focus, with vasoactive amines and intravenous antimicrobial agents being administered for 10 days (Meropenem combined with Fluconazole) according to the Campaign Surviving Sepsis and Latin America Sepsis Institute, ILAS.

The 840TM mechanical ventilator (Covidien-Nellcor and Puritan Bennett, Boulder, Colorado, USA) was used. The patient was tracheostomized after 48 hours of intubation, remaining for 25 days on mechanical ventilation in the ICU; after that, he was transferred to the prolonged ventilation unit after three unsuccessful attempts at conventional weaning trial with trach collar, in which he did not reach a spontaneous breathing higher than 15 minutes. At that time, the patient was awake, cooperative, hemodynamically stable, and free of infection. The IMT program using the electronic isokinetic loading device (POWERbreathe KH-2, London, UK) was started on day 26 of MV, in which 6 sets were done with 10 repetitions to achieve the 60 efforts daily intermittently and incrementally; see Figure 1.

**Figure 2:**
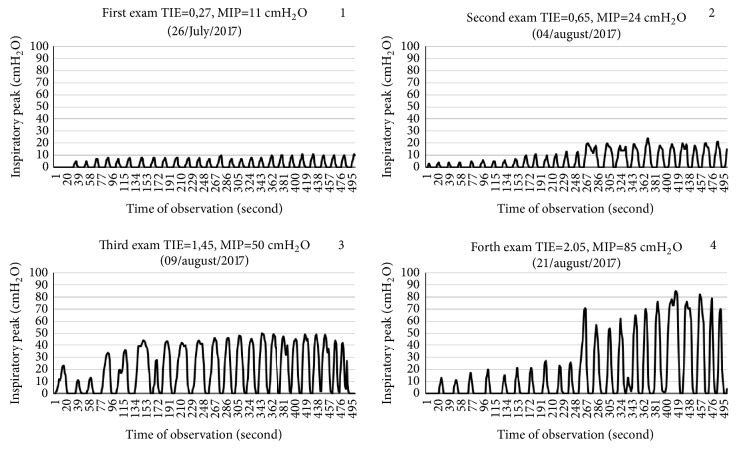
Graphics of inspiratory peaks in the serial weekly measurement of TIE index from the first until the last measurement along inspiratory muscle training program.

The muscle function of the patient at start of the training program evaluated by the Medical Research Council (MRC) scoring was 12 (20% of normal); the correspondent value using the functional status score (FSS-ICU) was 3 (8.6% of normal). By this time, his maximal inspiratory pressure (MIP) was 11cmH2O and the TIE index 0.27cmH_2_O/s, when the training load was adjusted to a maximum of 5 cmH_2_O (45% of the MIP). On day 36 of MV, his MIP had increased to 24 cmH_2_O with a TIE index of 0.65 cmH_2_O/s, a time when the training load was set to 12cmH_2_O (50% of the MIP). In the following week, the MIP reached 50cmH2O, the TIE index was 1.45 cmH_2_O/s, and the training load was set to 28cmH_2_O (56% of the MIP). On day 14 after the start of IMT, the patient was successfully weaned off the ventilator. A daily training with a load of 30cmH_2_O (60%) was maintained. On day 26 of IMT, his MIP was 85cmH_2_O and the TIE index 2.05cmH_2_O/s (Figure 2). He was discharged from the ICU after 64 days.

The IMT program was accompanied by an early mobilization and physical therapy protocol, active member exercising in bed followed by resistance exercising against gravity at the bedside, evolving to moving to standing position. Hospital discharge took place on September 8^th^, 2017, totaling 71 days of hospitalization. By that time, the scoring values using the MRC assessment and the FSS-ICU scale were 34 (57% of normal) and 18 (51.4 of normal), respectively.

## 4. Discussion

Prolonged MV is associated with several complications, such as ventilator-associated pneumonia (VAP), ventilation-induced diaphragmatic dysfunction (VIDD), and critical patient polyneuropathy [3, 6]. Jubram et al., in 2013, published a prospective, randomized, blind study in which 312 patients on prolonged ventilation (median stay in MV of 34 days) were analyzed. The success rate of weaning was 49%, patients who returned to mechanical ventilation after weaning success were 12.1%, hospital mortality rate was 55%, and mortality after 1 year was 63% [5].

Numerous efforts have been made in the attempt to reduce MV time, ICU length of stay, and sequel of immobility. For this purpose, some studies have used IMT with encouraging results. The use of IMT is associated with an increase in the success rate of prolonged weaning (71% compared to 47% in the control group) [7]. In a systematic review, in which 10 studies were selected, it was clear that only patients with difficulty in ventilatory weaning benefited from inspiratory muscle training [4]. The training of inspiratory muscles seems to emerge as an important adjunctive therapeutic tool to address the failure of weaning of long-stay patients in MV. However, it is still unclear which type of protocol should be used: load, duration, intensity, and frequency. Regarding this, the team experience with measurement of the TIE index was the only resource used as a guide to prescribe the IMT [2, 8].

The TIE index was idealized by Souza and Lugon (2015) and considers the combination of respiratory center stimulation and muscle response time. In two publications this index presented better accuracy than the other ones to predict the outcome of ventilatory weaning [2, 8]. It is our view that this new tool allows quantitative assessment of the inspiratory muscles supplanting the clinical judgment toward the guiding of the prescription of the parameters, which could help the rehabilitation process.

The score of MRC assessment at ICU admission was 12 (20% of normal) indicating severe muscle weakness. In contrast, at hospital discharge the score was 34 (57% of normal). Findings were similar when using the FSS-ICU scale, with corresponding numbers of 3 (8.6% of normal) and 18 (51.4% of normal). Our findings are in agreement with other reports suggesting that interventions that aim to encourage activity and early mobilization in the ICU may reduce duration of MV, length of stay in ICU, and at the same time improve patients' physical function [9, 10]. Only one study was found using the same electronic isokinetic inspiratory loading device and an increase in inspiratory muscle strength resulting in a favorable outcome was also reported [10]. However, caution should be exercised regarding conclusions as to the definite role of this new device in this setting because the experience with it is very small.

In conclusion, the use of inspiratory muscle training (IMT) with electronic isokinetic loading guided by serial measurement of the TIE index was found to be instrumental to provide strength gain and resistance to respiratory muscles. As a result, a patient with Guillain-Barré syndrome related to Zika virus infection was successfully weaned off the ventilator and discharged from the ICU and from the hospital. Of interest, at 6 months of hospital discharge, the patient remains on a regular physical therapy program and is gradually coming back to his normal days activities, like driving a car.
